# Association between serum vitamin D status and health-related quality of life (HRQOL) in an older Korean population with radiographic knee osteoarthritis: data from the Korean national health and nutrition examination survey (2010–2011)

**DOI:** 10.1186/s12955-015-0245-1

**Published:** 2015-04-18

**Authors:** Hye-Jung Kim, Jee-Yon Lee, Tae-Jong Kim, Ji-Won Lee

**Affiliations:** Department of Family Medicine, Severance Hospital, Yonsei University College of Medicine, 250 Seongsanno, Seodaemun-gu, Seoul, 120-752 Republic of Korea

**Keywords:** Vitamin D, Quality of life, EQ-VAS, EQ-5D, Osteoarthritis, knee

## Abstract

**Background:**

Vitamin D is important in bone health and its relationship with osteoarthritis has recently been reported. Both vitamin D deficiency and knee osteoarthritis are age dependent and are known to affect quality of life (QOL) in older populations. In this study, we aimed to determine the association between vitamin D status and health-related quality of life (HRQOL) in an older Korean population with knee osteoarthritis.

**Methods:**

A total of 2,165 participants aged ≥50 years with radiographic knee osteoarthritis defined as Kellgren-Lawrence (KL) grade ≥2 were selected from data from the 5th Korean National Health and Nutrition Examination Survey (KNHANES V), a representative cross-sectional nationwide survey conducted in 2010–2011. They stratified into two levels by vitamin D status (deficiency <10 ng/dL and normal ≥10 ng/dL). HRQOL was measured using the EuroQOL visual analogue scale (EQ-VAS) and the five dimensions and summary index of the EuroQOL-5 dimension (EQ-5D).

**Results:**

The vitamin D deficiency group was more likely to report problems with mobility, self-care, and usual activities. Vitamin D deficiency was significantly associated with poor HRQOL indicated by the lowest quartile of EQ-VAS (unadjusted odds ratio [OR] =1.832, p = 0.006) and the lowest quartile of the EQ-5D index (unadjusted OR = 1.992, p = 0.003). Theses associations of vitamin D status with EQ-VAS and EQ-5D index were maintained after adjustment for age and sex (Model 1: OR = 1.677, p = 0.022 and OR = 1.701, p = 0.021, respectively). The significant associations of vitamin D status with EQ-VAS were maintained after adjustment for other possible covariates (Model 3: OR = 1.562, p = 0.044). Also, a trend of associations between vitamin D status and EQ-5D index were shown after adjustment for other covariates (Model3: OR = 1.681, p = 0.056).

**Conclusion:**

This is the first study to reveal that vitamin D status is independently associated with HRQOL in an older Korean population with knee osteoarthritis. Our results suggest that the maintenance of sufficient vitamin D status may be important to prevent QOL decline in older populations with knee osteoarthritis.

**Electronic supplementary material:**

The online version of this article (doi:10.1186/s12955-015-0245-1) contains supplementary material, which is available to authorized users.

## Background

Vitamin D deficiency is a common problem affecting approximately 1 billion people worldwide [1,2], and elderly persons are more likely to experience vitamin D deficiency [3]. The basic role of vitamin D is the maintenance of calcium homeostasis and bone health [4]. Although the precise mechanisms remain unknown, vitamin D has recently been shown to be linked with various extraskeletal diseases, such as cardiovascular diseases, diabetes, cancer, auto-immune diseases, and depressive disorders [5-7]. Furthermore, because these chronic pathologic conditions are known to affect quality of life (QOL), vitamin D deficiency may also be related to QOL in old people. Many previous studies have assessed the relationships between vitamin D status and QOL in subjects with various medical conditions. Recent studies demonstrated the association between vitamin D and health-related QOL (HRQOL) in general older population [8,9]. However, these results were inconsistent in other groups of subjects with different medical conditions [10,11]. These inconsistencies resulted from the differences in methodologies, the association of vitamin D with specific diseases, study design, and study populations [12].

Osteoarthritis is the most common musculoskeletal disorder in older populations [13,14]. It is a major cause of chronic pain and physical disabilities and impairs HRQOL in older people [15,16]. Recently, new evidences are emerging that vitamin D deficiency is associated with osteoarthritis [4,17]. These results raise an expectation that vitamin D status may be a modifiable determinant to prevent QOL impairment in osteoarthritis. However, to our knowledge, no studies have specifically investigated the relationship between vitamin D status and HRQOL in older osteoarthritis patients.

Therefore, in the present study, we aimed to assess the association between vitamin D status and HRQOL in Korean elderly population with radiographically confirmed knee osteoarthritis using data from the first and second years (2010–2011) of the fifth Korean National Health and Nutrition Examination Survey (KNHANES V).

## Methods

### Study population

This cross-sectional study was based on data obtained from the first and second years (2010–2011) of the Korea National Health and Nutrition Examination Survey (KNHANES) V, a nationwide survey conducted by the Korea Center for Disease Control and Prevention. The survey represents the non-institutionalised civilian Korean population and consists of the Health Interview Survey and Health Examination Study. A multistage, stratified, probability sampling method was used, and households for sampling units were selected based on age, sex, and geographical area. Participants were informed that their household had been randomly selected to participate in this survey performed by the Korea Center for Disease Control and Prevention. They were given the right to refuse participation according to the National Health Enhancement Act supported by the National Statistics Law of Korea. All the participants signed an informed consent form.

Of the 17,476 participants in the KNHANES V conducted in 2010–1011, we excluded individuals without radiographic knee imaging and participants younger than 50 years. Because radiographic image studies were recommended to subjects aged 50 years and older, 6,327 participants underwent the radiographic examination for bilateral knee joints. After excluding 764 persons with missing data for blood vitamin D measurements (serum 25(OH)D levels), a total of 2,165 participants with radiographic knee osteoarthritis defined as Kellgren-Lawrence (KL) grade ≥2 were ultimately included in our analysis.

### Definition of radiographic knee osteoarthritis

Bilateral antero-posterior, lateral (30° flexion) and weight-bearing antero-posterior plain knee radiographs were taken using a SD 3000 Synchro Stand (Accele Ray SYFM Co., Seoul, Korea), and the radiographic images were reviewed by two radiologists. The degree of radiographic knee osteoarthritis was assessed according to the KL grading system: Grade 0, none: no visible features of OA; Grade 1, doubtful: questionable osteophytes or questionable joint space narrowing; Grade 2, minimal: definitive small osteophytes, minimal/mild joint space narrowing; Grade 3, moderate: definitive moderate osteophytes, joint space narrowing of at least 50%; Grade 4, severe: severely impaired joint space, subchondral bone cysts and sclerosis [18,19]. A subject who has radiographic osteoarthritis was defined if the KL grade was greater or equal to two [20].

### Demographic and behavioural variables and QOL

Demographic and behavioural variables included age (year), sex (male or female), residential region (urban or rural area), education level (middle school graduation or below), income (high or low, re-categorized from equivalized monthly household income expressed in quartiles). Marital status was divided into subjects who were married and lived with their spouse and subjects who were unmarried, widowed, divorced, or separated. For smoking status, subjects were classified into current smokers, ex-smokers, and non-smokers. The International Physical Activity Questionnaire (IPAQ) was adopted to measure physical activity frequency. Regular exercise was defined as participating in vigorous exercise (strenuous or gasping activities, such as running, high-speed cycling, and swimming) more than 3 times per week for ≥ 20 min at a time, moderate-intensity exercise (slightly strenuous or gasping activities such as slow swimming, badminton, and table tennis) more than 5 times per week for ≥ 30 min at a time, or walking more than 5 times per week for ≥ 30 min at a time. Subjects with a history of either hypertension, diabetes mellitus, dyslipidaemia, cerebrovascular accident, chronic renal disease, or liver cirrhosis were considered to have a chronic disease comorbidity. Completed questionnaires were reviewed by trained staff and entered in to a database.

Anthropometric measurements were performed by well-trained medical staff following a standardised protocol. Body mass index (BMI) was defined as weight divided by square of height (kg/m^2^). Waist circumference (cm) was measured at the umbilicus level with the subject standing and at the narrowest point between the lower border of the rib cage and the uppermost border of the iliac crest during normal expiration.

In KNHANES V, the EuroQOL-5 dimension (EQ-5D), EQ-5D index, and EuroQOL-visual analogue scale (EQ-VAS) developed by the EuroQOL group were used to measure HRQOL. EQ-5D consists of five questions to evaluate the level of self-reported problems in five dimensions (mobility, self-care, usual activities, pain or discomfort, and depression or anxiety), with three possible answers for each item (1 = no problem, 2 = moderate problem, 3 = severe problem) [21]. A summary index with a maximum score of 1 (EQ-5D index) can be calculated by a combination of each score of five dimensions and ranges from −0.171 to 1. The maximum score of 1 on EQ-5D index refer to the best health state with no problem in any of five dimensions. Participants described their subjective health status using EQ-VAS ranging from 0 (the worst imaginable health state) to 100 (the best imaginable health state).

### Vitamin D measurement

Blood samples were collected during the fasting state of health examination surveys. After collection, the samples were immediately refrigerated and transported to the designated central laboratory (NeoDin Medical Institute, Seoul, Korea). Serum 25(OH)D levels were measured using a radioimmunoassay kit (DiaSorin Inc., Stillwater, MN) with a 1470 WIZARD gamma–Counter (PerkinElmer, Waltham, MA). The serum 25(OH) D level represents the storage form of vitamin D, and the World Health Organization defined vitamin D deficiency and insufficiency as serum 25(OH)D levels below 25 nmol/L(10 ng/mL) and 50 nmol/L(20 ng/mL), respectively [22]. Also recent studies showed the strong association between vitamin D deficiency lower than 10 ng/mL and various pathologic conditions affecting QOL [23,24]. Therefore, vitamin D status was divided into two groups according to serum 25(OH) D concentration: deficiency (<10 ng/mL) and normal (≥10 ng/mL) referring to reference literatures [22,25].

### Statistical analysis

In all the analyses, the complex sampling and survey sample weights of the KNHANES were used to represent the entire Korean adult population without biased estimates. All data were presented as the estimated number, the weighted mean (SEs) for continuous variables, and the weighted proportions (% SEs) for categorical variables. The clinical characteristics of the study population between the vitamin D deficiency and vitamin D normal groups were compared using the generalised linear model for continuous variables and chi-square tests for categorical variables.

Chi-square tests were used to compare the prevalence of problems for each of the EQ-5 dimensions and quartiles of EQ-VAS and EQ-5D index according to vitamin D status. The scores in each of five problems were re-classified into two categories. EQ-VAS and EQ-5D index as continuous variables were divided into the quartiles defined as following QOL classification: 1st quartile (poor), 2nd quartile (fair), 3rd quartile (good), and 4th quartile (very good).

To investigate the association between vitamin D status and poor HRQOL in patients with radiographic knee osteoarthritis, the odds ratios (ORs) and 95% confidence intervals (CIs) for the prevalence of poor HRQOL based on vitamin D status were calculated after adjusting for covariates according to vitamin D status using multivariate logistic regression analysis. Before analysis, we classified participants EQ-5D index and EQ-VAS into quartiles. Then, the lowest quartiles of EQ-5D index and EQ-VAS were used as the indicators of poor HRQOL. Covariates included age, sex, body mass index (BMI), waist circumference (WC), education, income, residence, exercise, marital status, smoking status, comorbidity of chronic disease, and KL grade. They were the significant determinants (p value < 0.05) for poor HRQOL in univariate logistic regression analysis (data not shown) or clinically important variables on poor HRQOL identified in previous studies [26].

We performed all statistical analyses using the IBM Statistical Package for the Social Sciences, version 20.0 (SPSS Inc., Chicago, IL, USA). Statistical significance was defined as p < 0.05.

## Results

### Clinical characteristics according to vitamin D status in the study population

The subjects’ clinical characteristics according to vitamin D status are presented in Table 1. The number and weighted proportion of vitamin D deficiency group were 128 and 5.3%. The mean serum 25(OH)D levels were 8.45 ± 0.124 and 20.04 ± 0.296 ng/ml for the vitamin D deficiency and vitamin D normal groups, respectively. The vitamin D deficiency group was significantly older (69.02 ± 1.059 vs. 66.45 ± 0.286, p = 0.016) and showed a larger proportion of severe KL grade (35.4% vs. 19.5%, p < 0.0001) compared to the vitamin D normal group. In addition, the proportion of people who lived without their spouse was significantly higher in the vitamin D deficiency group (p = 0.017).

**Table 1:** **Clinical characteristics according to vitamin D status in the study population**

**Characteristics**	**Vit. D deficiency (<10 ng/mL)**	**Vit. D normal (≥10 ng/mL)**	**P value**
**N, sample size**	128	2037	
**%, weighted**	5.3%	94.7%	
**Mean vitamin D, ng/mL**	8.4 ± 0.124	20.04 ± 0.296	
**Age**			
Mean age, year	69.02 ± 1.059	66.45 ± 0.286	0.016^†^
**Gender**			
Male	24.1%	33.2%	0.104
Female	75.9%	66.8%	
**Body mass index (BMI)**			
Mean BMI, kg/m^2^	24.64 ± 0.457	24.79 ± 0.086	0.746
**Waist circumference (WC)**			
Mean WC, cm	84.07 ± 1.364	85.44 ± 0.272	0.317
**Education**			
≤Elementary school graduate	71.5%	62.2%	0.096
≥Middle school graduate	28.5%	37.8%	
**Income**			
1st, 2nd quartile (Low income)	66.7%	64.6%	0.704
3rd, 4th quartile (High income)	33.3%	35.4%	
**Residence**			
Urban	73.0%	65.5%	0.222
Rural	27.0%	34.5%	
**Marital status**			
Without spouse (Unmarried, or widowed/divorced/separated)	42.2%	30.1%	0.017^†^
Married, With spouse	57.8%	69.9%	
**Smoking**			
Never	72.4%	65.9%	0.482
Ex-smoker	15.3%	20.6%	
Current smoker	12.3%	13.5%	
**Regular exercise** ^‡^			
No	61.7%	52.0%	0.100
Yes	38.3%	48.0%	
**Comorbidity of chronic disease** ^§^			
No	40.8%	45.8%	0.314
Yes	59.2%	54.2%	
**Kellgren-Lawrence grade**			
grade 2 (mild)-3 (moderate)	64.6%	80.5%	<0.0001^†^
grade 4 (severe)	35.4%	19.5%	

Data are presented as weighted proportions unless otherwise indicated. Continuous results presented as weighted mean with standard error.

^†^Statistically significant p-value ≤ 0.05. P-values were determined by chi-square test for categorical variables and t-test for continuous variables.

^‡^Regular exercise was defined as participating in vigorous exercise (strenuous or gasping activities, such as running, high-speed cycling, and swimming) more than 3 times per week for ≥20 min at a time or moderate-intensity exercise (slightly strenuous or gasping activities, such as slow swimming, badminton, and table tennis) more than 5 times per week for ≥30 min at a time, or walking more than 5 times per week for ≥30 min at a time.

^§^Comorbidity of chronic disease included subjects with hypertension, diabetes, dyslipidaemia, cerebrovascular accident, chronic renal disease, or liver cirrhosis.

*Abbreviations*: BMI, body mass index; Vit. D, vitamin D; WC, waist circumference.

### Comparison of the EQ-5 dimensions, EQ-VAS, and EQ-5D index according to vitamin D status

The weighted proportions of problems for each of EQ-5 dimensions and each quartile of EQ-VAS and EQ-5D index according to vitamin D status are presented in Table 2. The proportion of people having problems with mobility, self-care, and usual activity was significantly higher in the vitamin D deficiency group than the vitamin D normal group (56.1% vs. 42.7%, p = 0.007, 19.3% vs. 10.3%, p = 0.014, and 38.4% vs. 24.4%, p = 0.005, respectively). In addition, these differences were still remained when we re-analyzed among subdivided vitamin D groups such as vitamin D deficiency (<10 ng/mL), insufficiency (10-20 ng/mL), and sufficiency (>20 ng/mL).The significant differences were presented between vitamin D deficiency and insufficiency group rather than between vitamin D insufficiency and sufficiency groups (56.1% vs 42.8% vs 42.5%, p = 0.049, 19.3% vs 10.9% vs 9.5%, p = 0.028, and 38.4% vs 25.1% vs. 23.6%, p = 0.026, respectively) (Additional file 1). However, there was no significant difference in the proportion of people with problems in pain/discomfort or depression/anxiety among vitamin D subgroups. Furthermore, the proportions of vitamin D deficient participants in the 1st (lowest) quartiles of EQ-VAS and EQ-5D index defined as poor HRQOL were significantly higher compared with the vitamin D normal group (35.2% vs. 22.9%, p = 0.011 and 37.7% vs. 23.3%, p = 0.006, respectively) (Table 2) and compared with the vitamin D insufficiency and sufficiency groups (35.2% vs. 22.7% vs. 23.2%, p = 0.040 and 37.7% vs. 23.8% vs. 22.8%, p = 0.114, respectively) (Additional file 1).

**Table 2:** **Comparison of the EQ-5 Dimensions, EQ-VAS, and EQ-5D index according to vitamin D status**

**Health-related QOL**		**Vit. D deficiency (<10 ng/mL)**	**Vit. D normal (≥10 ng/mL)**	**P value**
**EQ-5Dimensions**				
Problem with mobility	None	43.9%	57.3%	0.007^†^
	Some or severe	56.1%	42.7%	
Problem with self-care	None	80.7%	89.7%	0.014^†^
	Some or severe	19.3%	10.3%	
Problem with usual activity	None	61.6%	75.6%	0.005^†^
	Some or severe	38.4%	24.4%	
Problem with pain/discomfort	None	59.2%	60.3%	0.827
	Some or severe	40.8%	39.7%	
Problem with depression/anxiety	None	79.1%	84.4%	0.273
	Some or severe	20.9%	15.6%	
**EQ-VAS** ^**‡**^	Mean ± SE			
1st quartile	38.90 ± 0.899	35.2%	22.9%	0.011^†^
2nd quartile	62.40 ± 0.351	27.2%	28.9%	
3rd quartile	78.13 ± 0.189	25.7%	22.6%	
4th quartile	92.69 ± 0.295	11.9%	25.6%	
**EQ-5D index** ^**‡**^	Mean ± SE			
1st quartile	0.62 ± 0.009	37.7%	23.3%	0.006^†^
2nd quartile	0.84 ± 0.002	26.1%	26.9%	
3rd quartile	0.91 ± 0.000	3.1%	6.1%	
4th quartile	1.00 ± 0.000	33.1%	43.7%	

Data are presented as weighted proportions unless otherwise indicated. Continuous results presented as weighted mean with standard error.

^†^Statistically significant p-value ≤ 0.05. P-values were determined by chi-square test.

^‡^The quartiles of EQ-VAS and EQ-5D index were defined as the following QOL classification: 1st quartile (poor), 2nd quartile (fair), 3rd quartile (good), 4th quartile (very good).

*Abbreviation*: EQ-5D, EuroQOL-5 dimension; EQ-VAS, EuroQOL-visual analogue scale; QOL, quality of life; SE, standard error; Vit. D, vitamin D.

### Prevalence of poor HRQOL based on vitamin D status

To assess the association between vitamin D deficiency and poor HRQOL, we compared the prevalence of poor HRQOL (the first quartile of EQ-VAS and EQ-5Dindex) based on vitamin D status. The odds ratios(OR) and 95% confidence intervals (95% CI) for the prevalence of poor HRQOL in vitamin D deficiency compared to vitamin D normal group are shown in Table 3. In the unadjusted analysis, vitamin D deficiency was significantly associated with the first quartile of EQ-VAS with an OR of 1.832 (95% CI 1.187–2.827, p = 0.006) and the first quartile of EQ-5D index with an OR of 1.992 (95% CI 1.266–3.132, p = 0.003). The multivariate OR for the low EQ-VAS and the low EQ-5D index comparing the vitamin D deficiency with vitamin D normal groups were 1.677 ( p = 0.022) and 1.701 (p = 0.021) after adjustment for age and sex which were important demographic factors in HRQOL(Model1). The multivariate ORs for the low EQ-VAS and EQ-5D index as poor HRQOL indices in vitamin D deficiency group versus vitamin D normal group were 1.585 (p = 0.041) and 1.485(p = 0.103), respectively, after adjustment for marital status and KL grade which showed significantly different characteristics by vitamin D status in addition to age and sex in Model 2. Although statistical significance were not remained in EQ-5D index, they still showed a trend of higher prevalence of poor HRQOL in vitamin D deficiency group (OR 1.618, p = 0.056) after combined adjustment for other covariates (Model3). Furthermore, the multivariate ORs for the lowest quartile of EQ-VAS in vitamin D deficiency group versus vitamin D normal group was 1.562 (p = 0.044) after combined adjustment for other possible covariates (Model3).

**Table 3:** **Prevalence of poor HRQOL based on vitamin D status**

	**Unadjusted**		**Model 1**		**Model 2**		**Model 3** ^**‡§**^	
	**Odds ratio (95% CI)**	**P value**	**Odds ratio (95% CI)**	**P value**	**Odds ratio (95% CI)**	**P value**	**Odds ratio (95% CI)**	**P value**
**EQ-VAS**								
Vit. D normal	Reference		Reference		Reference		Reference	
Vit. D deficiency	1.832 (1.187-2.827)	0.006^†^	1.667 (1.077-2.580)	0.022^†^	1.585 (1.019-2.465)	0.041^†^	1.562 (1.011-2.413)	0.044^†^
**EQ-5D index**								
Vit. D normal	Reference		Reference		Reference		Reference	
Vit. D deficiency	1.992 (1.266-3.132)	0.003^†^	1.701 (1.083-2.670)	0.021^†^	1.485 (0.922-2.392)	0.103	1.618 (0.988-2.650)	0.056

To see the association between vitamin D deficiency and poor HRQOL, we compared the prevalence of poor HRQOL (the first quartile of EQ-VAS and EQ-5D index) based on vitamin D status. Vitamin D status were classified into 2 groups according to serum 25(OH)D concentration : Vitamin D normal (≥10 ng/mL) and deficiency(<10 ng/mL).

^†^Statistically significant p-value ≤ 0.05. P-values were determined by multivariate logistic regression analysis.

Model1: Adjusted for age & gender.

Model2: Adjusted for age, gender + marital status, KL grade.

Model3: Adjusted for age, gender + marital status, KL grade + education, income, residence, smoking, regular exercise^‡^, comorbidity of chronic diseases^§^, BMI, WC.

^‡^Regular exercise was defined as participating in vigorous exercise (strenuous or gasping activities, such as running, high-speed cycling, and swimming) more than 3 times per week for ≥20 min at a time or moderate-intensity exercise (slightly strenuous or gasping activities, such as slow swimming, badminton, and table tennis) more than 5 times per week for ≥30 min at a time, or walking more than 5 times per week for ≥30 min at a time.

^§^Comorbidity of chronic disease included subjects with hypertension, diabetes, dyslipidaemia, cerebrovascular accident, chronic renal disease, or liver cirrhosis.

*Abbreviation*: CI, confidence interval; BMI, body mass index; EQ-5D, EuroQOL-5 dimension; EQ-VAS, EuroQOL-visual analogue scale; HRQOL, health-related quality of life; KL grade, Kellgren-Lawrence grade; QOL, quality of life; Vit. D, vitamin D; WC, waist circumference.

Additionally, vitamin D deficiency group showed a significant association with poor HRQOL indices such as EQ-VAS (OR = 2.360, p = 0.024 in Model 1, OR = 2.291, p = 0.035 in Model 2, and OR = 1.934, p = 0.137 in Model 3) and EQ-5D index (OR =2.390, p = 0.031 in Model 1, OR = 2.324, p = 0.043 in Model 2, and OR = 2.803, p = 0.021 in Model 3) when we re-analyzed after excluding participants comorbid with chronic diseases to control the impact of other chronic diseases completely (Table 4). Their sample size were 903(95.3%) for vitamin D normal group and 46(4.7%) for vitamin D deficiency group, respectively.

**Table 4:** Prevalence of poor HRQOL based on vitamin D status after excluding participants comorbid chronic disease*

	**Unadjusted**		**Model 1**		**Model 2**		**Model 3** ^**‡**^	
	**Odds ratio (95% CI)**	**P value**	**Odds ratio (95% CI)**	**P value**	**Odds ratio (95% CI)**	**P value**	**Odds ratio (95% CI)**	**P value**
**EQ-VAS**								
Vit. D normal	Reference		Reference		Reference		Reference	
Vit. D deficiency	2.253 (1.037-4.896)	0.040^†^	2.360 (1.120-4.976)	0.024^†^	2.291 (1.059-4.955)	0.035^†^	1.934 (0.810-4.617)	0.137
**EQ-5D index**								
Vit. D normal	Reference		Reference		Reference		Reference	
Vit. D deficiency	2.118 (0.942-4.762)	0.069	2.390 (1.085-5.266)	0.031^†^	2.324 (1.028-5.251)	0.043^†^	2.803 (1.172-6.703)	0.021^†^

To control the impact of other chronic diseases, we compared the prevalence of poor HRQOL (the first quartile of EQ-VAS and EQ-5Dindex) based on vitamin D status after excluding participants comorbid chronic disease*. Vitamin D status were classified into two groups according to serum 25(OH)D concentration: Vitamin D normal (≥10 ng/mL) and deficiency (<10 ng/mL).

*Participancts comorbid chronic disease included subjects with hypertension, diabetes, dyslipidaemia, cerebrovascular accident, chronic renal disease, or liver cirrhosis.

^†^Statistically significant p-value ≤ 0.05. P-values were determined by multivariate logistic regression analysis.

Model1: Adjusted for age & gender.

Model2: Adjusted for age, gender + marital status, KL grade.

Model3: Adjusted for age, gender + marital satus, KL grade + education, income, residence, smoking, regular exercise^‡^, BMI, WC.

^‡^Regular exercise was defined as participating in vigorous exercise (strenuous or gasping activities, such as running, high-speed cycling, and swimming) more than 3 times per week for ≥20 min at a time or moderate-intensity exercise (slightly strenuous or gasping activities, such as slow swimming, badminton, and table tennis) more than 5 times per week for ≥30 min at a time, or walking more than 5 times per week for ≥30 min at a time.

*Abbreviation*: CI, confidence interval; BMI, body mass index; EQ-5D, EuroQOL-5 dimension; EQ-VAS, EuroQOL-visual analogue scale; HRQOL, health-related quality of life; KL grade, Kellgren-Lawrence grade; QOL, quality of life; Vit. D, vitamin D; WC, waist circumference.

## Discussion

In this nationwide cross-sectional study, we examined a possible association between vitamin D status and HRQOL using a representative sample of an older Korean population (≥50 years) with radiographically confirmed knee osteoarthritis. We found that vitamin D deficiency was associated with poor QOL independently of other covariates and possible confounders, including age, sex, BMI, waist circumference, education, income, residence, exercise, marital status, smoking status, comorbidity of chronic disease, and KL grade, in an older Korean population with knee osteoarthritis.

Recently, a role of vitamin D in QOL was identified in various disease groups, as well as the general population. However, many results are inconclusive and often contradictory [10,11,27-29]. The methodologic inconsistencies for measurement of vitamin D and HRQOL, differences in study design, and the great heterogeneity in study populations (healthy vs diseased) would make it difficult to draw conclusions [12]. To the best of our knowledge, this is the first study that investigated the association between vitamin D and HRQOL limited in an older population with knee osteoarthritis.

The prevalence of vitamin D deficiency in our study population was 5.3% lower than other studies. These may resulted from a different definition of vitamin D deficiency and different study populations. Also, inter-method variability in 25(OH)D measurements seems to contribute to this result [30,31]. Therefore, standardization of methods for the quantification of 25(OH)D on a human-based sample panel would be required to reduce the inter-method variability and establish reference values for 25(OH)D in further studies.

Although we could not determine causality with our cross-sectional study, we suggest three possible underlying mechanisms. First, comparing clinical characteristics according to vitamin D status, the vitamin D deficiency group included a higher proportion of subjects with the severe form of radiographic osteoarthritis (higher KL grade). This finding is in line with previous studies showing that low vitamin D levels were associated with structural changes and progression of radiographic knee osteoarthritis [16]. Although the pathogenesis of osteoarthritis is not clear, recent studies suggested that an imbalance of the subchondral bone remodeling may initiate the degenerative change [32]. Vitamin D deficiency may contribute to this degenerative change in such a way that the decrease in serum 25(OH)D concentration accelerates osteoclastogenesis and bone resorption [1]. Therefore, vitamin D deficiency may aggravate pathologic knee osteoarthritis changes, and this may be one mechanism to explain HRQOL impairment in this population.

Muscular weakness may be another physiological explanation for the association between vitamin D deficiency and impaired QOL. Several previous studies demonstrated that vitamin D supplementation reduced the risks of falls and fractures, and these results were explained by the beneficial effect of vitamin D on muscle strength, balance, and bone health in older populations [33,34]. These findings were partly consistent with other studies in subjects with knee osteoarthritis [35]. Furthermore, muscular weakness is a well-known risk factor for decreased QOL in older populations with and without knee osteoarthritis [36-38]. Although the precise relationship between muscular weakness and QOL is not fully understood, declines in mobility [39] and daily activity performance [40] due to muscular weakness is considered to contribute to impaired QOL in elderly subjects. We found that the vitamin D deficiency group showed a higher proportion of problems with mobility and usual activities compared with the vitamin D normal group, which is in line with previous studies reporting a positive association of vitamin D with muscle strength and physical performance [35,41]. Therefore, muscular weakness induced by vitamin D deficiency may affect HRQOL in this population.

The third possible physiological mechanism that could explain the relationship between vitamin D deficiency and poor QOL is impairment of self-care and usual activities related to cognitive function. A recent study revealed that vitamin D levels were associated with cognitive function, which is an important factor affecting QOL in older people with chronic conditions [41]. Furthermore, previous studies have reported significant declines of self-care [42] and usual activities in patients with cognitive impairment [43,44]. Although we could not evaluate cognitive function in our study population, our results showed significant impairment of self-care and usual activities in the vitamin D deficiency group compared with the control group. Therefore, it is possible to hypothesise that problems with self-care and usual activities related to cognitive impairment may affect the QOL in population with knee osteoarthritis. Further prospective interventional studies that assess cognitive function are needed to investigate the precise role of cognitive function in association with vitamin D and QOL in osteoarthritis patients.

Unlike several previous studies [9,45], we did not find a difference in the proportion of subjects with pain/discomfort and depression/anxiety between the vitamin D deficiency and normal groups. These inconsistencies may result from an underestimation for problems with ‘pain or discomfort’ in subjects with knee osteoarthritis due to their use of analgesics, small sample size in vitamin D deficiency group, and different populations. Therefore, further studies are necessary to assess the contribution of vitamin D status to physical and mental components of QOL in a large number of subjects with knee osteoarthritis.

Our study has several limitations. First, this was a cross-sectional study and is thus unable to establish a causal relationship between vitamin D and HRQOL. Second, although we adjusted for possible confounding covariates, we acknowledge that other factors may have affected the association between vitamin D and HRQOL because of inadequacies in data use. For example, the nutrition status, sun exposure, and comorbidity of osteoporosis were not included, despite their known influences on vitamin D status and HRQOL [46-48]. The third limitation is related to the use of self-reported questionnaires that are prone to error, even though the EQ-5D and EQ-VAS are well-established, validated instruments.

Despite these limitations, this is the first study to investigate the association between vitamin D and the five HRQOL dimensions in an older population with knee osteoarthritis using population-based data. We suggested that vitamin D measurement at the time of diagnosing osteoarthritis and vitamin D supplementation for vitamin D deficient patients with knee osteoarthritis would be considered to prevent the QOL decline if well-founded studies have been collected.

## Conclusion

In conclusion, vitamin D status was independently associated with HRQOL in an older Korean population with knee osteoarthritis. Although we could not determine causality, our results collectively suggested that the maintenance of sufficient vitamin D status may be important to prevent QOL decline in an older population with knee osteoarthritis. Further studies to investigate a possible causal relationship and to clarify the precise mechanisms underlying the association of vitamin D with HRQOL should be performed by randomized controlled trials and cohort studies. Additionally, it is necessary to determine the sufficient level of vitamin D considering the effects of nutritional status and to identify other nutritional factors potentially affecting HRQOL in those with knee osteoarthritis.

## Additional file

Additional file 1:
**Comparison of the EQ-5 Dimensions, EQ-VAS, and EQ-5D index among vitamin D subgroups.**
